# When less is more: non-monotonic spike processing in neurons

**DOI:** 10.1186/1471-2202-14-S1-P389

**Published:** 2013-07-08

**Authors:** Hinrich Arnoldt, Sven Jahnke, Lena Happ, Jannes Gladrow, Birk Urmersbach, Shuwen Chang, Holger Taschenberger, Marc Timme

**Affiliations:** 1 Network Dynamics Group, Max Planck Institute for Dynamics and Self-Organization, Göttingen, Germany; 2 Bernstein Center for Computational Neuroscience Göttingen, Göttingen, Germany; 3 Georg-August-Universität Göttingen, Göttingen, Germany; 4 Membrane Biophysics Department, Max Planck Institute for Biophysical Chemistry, Göttingen, Germany

## 

**Figure 1:**
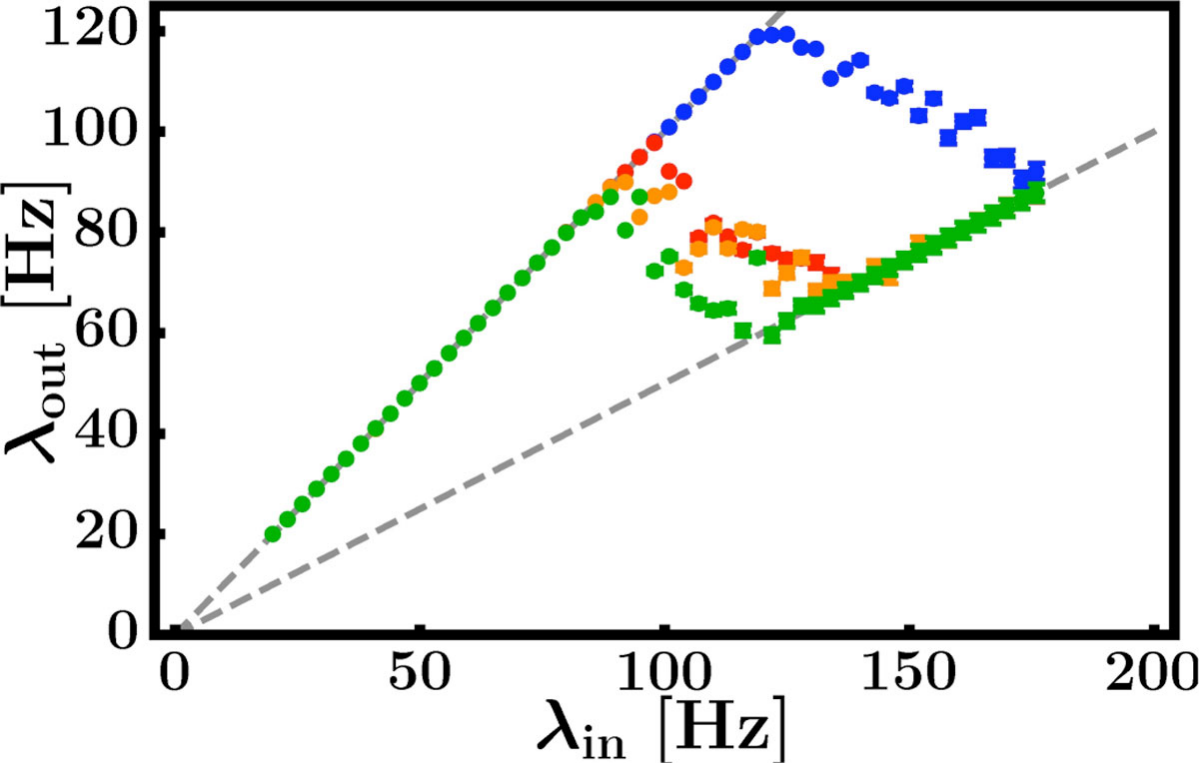
**Non-monotonic response to regular spiking inputs as observed in neurons from the medial nucleus of the trapezoid body (MNTB) of rats**. Shown is the measured output rate in dependence of the input rate of the neuron. Dashed lines indicate theoretical predictions (parameter-free), colored data results from experiment. Different colors code for different input strength of the spiking input.

Most neurons in the nervous system communicate by sending and receiving stereotyped electrical pulses called action potentials or spikes. The computational capabilities of neural circuits centrally rely on the input-output relations of single neurons. This relation is commonly characterized by its output spike rate in response to a temporally continuous constant input current, sometimes in the presence of additional current fluctuations [1]. If the spikes each neuron receives are irregular in time and individually only weakly affect the neuron's membrane potential, this continuous-input picture serves as an appropriate approximation [2] to the actual spike sequence input. As a consequence, a neuron's response curve in terms of its output spike rate, increasing monotonically as a function of input current, is considered one of its most important standard characteristics. Yet, a broad range of neural systems exhibit more regular, patterned spike sequences. Some experimental and numerical studies [3-5] suggest that certain neurons receiving correlated spiking inputs may exhibit non-monotonic input-output relations. Here we systematically analyze the stationary spiking response of neurons to regular spiking inputs and reveal that it is generically non-monotonic. Our theoretical analysis shows that the underlying mechanism relies solely on a combination of the discrete nature of the communication by spikes and limited resources required for spike processing. Numerical simulations of mathematically idealized and biophysically detailed models, as well as neurophysiological experiments confirm our theoretical predictions (Figure 1). Non-monotonic response to regular spiking inputs thus is a generic feature common across spiking neurons.
